# Deciphering *Prunus* Responses to PPV Infection: A Way toward the Use of Metabolomics Approach for the Diagnostic of Sharka Disease

**DOI:** 10.3390/metabo11070465

**Published:** 2021-07-19

**Authors:** Christian Espinoza, Benoît Bascou, Christophe Calvayrac, Cédric Bertrand

**Affiliations:** 1 PSL Université de Paris EPHE-UPVD-CNRS, USR 3278 CRIOBE, Université de Perpignan Via Domitia, 52 Avenue Paul Alduy, CEDEX, 66860 Perpignan, France; christian.espinoza@etudiant.univ-perp.fr (C.E.); benoit.bascou@etudiant.univ-perp.fr (B.B.); 2 S.A.S. AkiNaO, Université de Perpignan Via Domitia, 52 Avenue Paul Alduy, CEDEX, 66860 Perpignan, France; 3 Biocapteurs-Analyses-Environnement, Université de Perpignan Via Domitia, 52 Avenue Paul Alduy, CEDEX, 66860 Perpignan, France; christophe.calvayrac@univ-perp.fr; 4 Laboratoire de Biodiversité et Biotechnologies Microbiennes, USR 3579 Sorbonne Universités (UMPC) Paris 6 et CNRS, Observatoire Océanologique, Banyuls-sur-Mer, CEDEX, 75005 Paris, France

**Keywords:** *Plum pox virus*, *Prunus*, metabolomics

## Abstract

Sharka disease, caused by *Plum pox virus* (PPV), induces several changes in *Prunus*. In leaf tissues, the infection may cause oxidative stress and disrupt the photosynthetic process. Moreover, several defense responses can be activated after PPV infection and have been detected at the phytohormonal, transcriptomic, proteomic, and even translatome levels. As proposed in this review, some responses may be systemic and earlier to the onset of symptoms. Nevertheless, these changes are highly dependent among species, variety, sensitivity, and tissue type. In the case of fruit tissues, PPV infection can modify the ripening process, induced by an alteration of the primary metabolism, including sugars and organic acids, and secondary metabolism, including phenolic compounds. Interestingly, metabolomics is an emerging tool to better understand *Prunus*–PPV interactions mainly in primary and secondary metabolisms. Moreover, through untargeted metabolomics analyses, specific and early candidate biomarkers of PPV infection can be detected. Nevertheless, these candidate biomarkers need to be validated before being selected for a diagnostic or prognosis by targeted analyses. The development of a new method for early detection of PPV-infected trees would be crucial for better management of the outbreak, especially since there is no curative treatment.

## 1. Introduction

Sharka disease, caused by PPV, is one of the most devastating viral diseases of the genus *Prunus*. PPV is a member of the genus *Potyvirus* in the family *Potyviridae*. In 1932, Atanasoff described sharka as a viral disease, and in 1917 to 1918, the first cases of sharka were reported in Bulgaria [1]. Ten strains of PPV, clearly differentiated from the molecular, serological, and evolutionary perspectives, have been recognized: PPV-D (Dideron), PPV-M (Marcus), PPV-EA (El Amar), PPV-C (Cherry), PPV-Rec (Recombinant), PPV-W (Winona), PPV-T (Turkey), PPV-CR (Cherry Russian), PPV-AM (Ancestor Marcus), and PPV-CV (Cherry Volga) [2].

Sharka symptoms may appear on bark, petals, shoots, leaves, fruit, and stones. Most of the susceptible cultivars show yellowish-green rings, mosaic mottling, and distortions on the leaves. Infected fruits may drop prematurely, deform, or display chlorotic spots and necrotic areas, making them unsuitable for consumption or industrial processing [3]. Apricots, European plums, peaches, and Japanese plums are particularly affected by PPV infection, resulting in reduced yields and fruit qualities. Sometimes, losses reach 100% in susceptible cultivars. The costs associated with sharka disease, including direct and indirect costs, are estimated at 10 billion Euros worldwide over 30 years [1].

PPV spreads over long distances by uncontrolled movements of infected plant materials. The transmission is possible by the grafting and vegetative multiplication of infected plants. Subsequently, natural and local spreads occur by many species of aphid, which transmit the virus in a nonpersistent manner [1]. Furthermore, pesticides do not act fast enough to prevent the spread of the virus in orchards. Currently, sharka is present worldwide and causes severe economic losses in susceptible cultivars [3].

The development of resistant cultivars and rootstocks remains one of the most ecologically and economically suitable approaches to achieve a long-term control of sharka disease. Nevertheless, few natural sources of resistance to PPV have been found in *Prunus* species [4]. These sources of resistance have been found in apricots, almonds, plums, and in a wild relative of peaches, *Prunus davidiana*. Still, no known source of resistance has been identified in peaches [1].

To date, while there is no known treatment for PPV, a variety of different management strategies have been applied to deal with sharka disease [3]. For example, in France, the focus is placed on limiting the disease through: (i) surveillance of nurseries and orchards, (ii) the removal of infected plants, and (iii) the use of PPV-free plant materials. Surveillance methods rely on visual inspections of *Prunus* orchards to detect sharka symptoms combined with leaf sampling followed by serological or molecular diagnostic tests. However, a visual inspection does not guarantee the sanitary status of the tree [3,5]. In several cases, the use of analytical methods is necessary. Diagnostic tests directly detect the presence of PPV in plant materials, and some can even identify the strain of PPV. These techniques are divided into three groups: (i) biological indexing on susceptible *Prunus* indicator plants, (ii) serological tests, and (iii) molecular tests. However, the irregular distribution of PPV in the tree and the seasonal variation in the viral load may limit the diagnostic accuracy [3]. To minimize these limitations, the sampling procedures have been optimized [6]. In addition, despite these reliable methods for PPV detection, no current method can detect PPV during the latency period [3].

Interestingly, under controlled conditions, it has been shown that there exists a mismatch between the incubation and the latency periods, with the latter lasting a day longer. Under field conditions, this mismatch could be even more important. Thus, while all infectious trees could hypothetically be detected by visual detection, this would be not economically feasible on a daily basis. Therefore, the delay between the appearance of symptoms and the detection of these trees promotes the spread of PPV [7]. Moreover, in *Prunus* species, the PPV incubation period can range from a few months to a few years. This period may depend on several viral and host parameters [3].

The development of a new approach to detect and identify the metabolic responses of PPV-infected trees during the incubation period could be very promising for the management of PPV propagation. The approach consists of early detection to ensure the removal of infected trees before the end of the latent period, i.e., before the infectious state. It can be assumed that PPV-infected trees respond upon infection, inducing stress or defense responses. In addition, the existence of systemic responses to PPV infection, i.e., in symptomatic or asymptomatic plant materials, is of great interest. This could lead to the development of a reliable and efficient approach to detect infected trees, despite the heterogeneous distribution of the virus in plants.

Deciphering *Prunus*–PPV interactions could provide answers to these questions. It could support the hypothesis for the existence of an early response to the onset of symptoms, and on the other hand, it could argue for the existence of a systemic response to the infection. Moreover, metabolomics appears to be a promising approach for the study of plant–pathogen interactions. Through a nontargeted analysis, it provides a global profile of the plant metabolome and, thus, highlights the main alterations caused by the pathogen [8].

In this review, we thus focus only on the changes induced by PPV in *Prunus* species. To date, most of these studies have presented impacts on leaves, including the chloroplast, antioxidant system, phytohormones, proteome, transcriptome, and translatome. Further, the vast majority of recent studies on fruits have concentrated on primary and secondary metabolisms. We also discuss the potential of the metabolomics approach for the study of *Prunus*–PPV interactions and the development of a new method for the detection of PPV-infected trees. We organized this paper into four sections: the impact of PPV on (i) the leaves of susceptible varieties, (ii) the leaves of resistant varieties, (iii) the fruit of susceptible varieties, and (iv) the usefulness of the metabolomics approach for the study of *Prunus*–PPV interactions and for the detection of infected trees.

## 2. How Does the *Plum Pox Virus* Affect the Susceptible *Prunus* Leaves?

In this section, we focus primarily on the modifications produced by PPV on leaves that display sharka symptoms on susceptible peach (GF-305), apricot (Real Fino/Z506-7/Canino), and plum (European plum) varieties. In the majority of the studies described here, the Dideron strain was used to infect trees.

### 2.1. The Chloroplast: The Organelle Most Affected by PPV Infection

At the cellular level, the PPV infection produced ultrastructure alterations in both susceptible peaches (GF-305) and apricots (Real Fino). Several authors have largely emphasized that, in peaches, the viral infection affects the thylakoids, plastoglobulus, granal, and starch content, indicating that the chloroplast is the most affected cell organelle [9,10,11]. Dilated thylakoid membranes with a reduced amount of grana, an increase in the number and size of plastoglobuli, and a decrease in starch content in chloroplasts were observed by Hernández et al. [9]. The alteration in the starch content seems to be localized in chloroplasts from palisade parenchyma. In addition, Clemente-Moreno et al. [10] described that PPV induced a disorganized granal structure. In apricots, PPV infection also led to a significant modification in the chloroplast ultrastructure, resulting in the dilatation of thylakoid membranes and in the absence of starch grains in the chloroplasts, while the other organelles remained unaffected [12]. However, these authors also demonstrated that physiological responses appear to be different in apricots and peaches. For instance, the percentage of chloroplasts that showed dilated thylakoids in susceptible apricot plants was lower than that observed for PPV-infected peach plants.

In addition, many authors have observed that PPV infections produce alterations in the chlorophyll fluorescence parameters in both susceptible peaches (GF-305) and apricots (Real Fino) [9,10,12,13]. Chlorophyll molecules absorb light energy, which is then dissipated in three competitive ways: (i) photosynthesis, (ii) heat dissipation, and (iii) chlorophyll fluorescence. The measurement of chlorophyll fluorescence parameters can therefore be used to assess the heat dissipation and, in particular, the variation of photosynthesis activity [13]. In general, several chlorophyll fluorescence parameters revealed alterations of the photosynthesis apparatus, including the PSII and the electron transport in PSII [9,10,12,13]. Therefore, the alteration of the photosynthesis provoked by a PPV infection could affect the primary metabolism. In addition, heat dissipation also appeared to be affected by the PPV infection [9]. This process is responsible for removing excess excitation energy in plants and for preventing the accumulation of harmful free radicals [13,14]. Thus, the drop in this process could indicate photooxidative damage and the production of harmful species, such as singlet oxygen (^1^O_2_) [9]. In conclusion, the variation in the chlorophyll fluorescence parameters in both peaches and apricots could reveal an alteration of PSII functioning, as well as higher reactive oxygen species (ROS) generation, in chloroplasts.

Changes in the chlorophyll fluorescence parameters and cell ultrastructure reflected an alteration of the photosynthetic apparatus and chloroplast metabolism. Moreover, the disorganized chloroplast structure could be the result of a direct effect of virus infection on photosynthesis by the induction of photoinhibitory damage in the reaction centers and the increase in ROS generation in the chloroplasts. ROS accumulation can affect the membrane components, which results in the disorganization of the chloroplast structure and which then reveals oxidative stress in chloroplasts. Furthermore, an increase in the ROS levels is accompanied by an increase in electrolyte leakage, lipid peroxidation, protein peroxidation, and the appearance of oxidative microbursts [12,15,16]. It is important to note that chlorophyll fluorescence parameters, chloroplast ultrastructure alterations, and H_2_O_2_ accumulation were more highly altered in peach leaves than in those observed in susceptible apricots. These differences could be explained by the fact that peach plants are more susceptible to PPV infection and show more chlorosis symptoms than the apricot cultivar Real Fino. Although there are a significant number of studies in the literature on the physiological alterations and damage to *Prunus* caused by PPV infection, it should be noted that some conflicting results have been reported. The lack of clarity is probably due to a combination of variables, including metabolic differences between the plant species studied, differences in the process of infection, the level of PPV infection, the conditions under which the plants were grown, and the time of sampling.

### 2.2. Major Changes in the Antioxidant System as a Result of PPV Infection in Susceptible Varities

We previously summarized that PPV infection is caused an oxidative stress in chloroplasts by ROS accumulation. The antioxidant system, which includes enzymatic and nonenzymatic defense systems, is responsible for maintaining the balance of ROS detoxification and production. The first defense mechanism in plants is the enzyme superoxide dismutase (SOD), which involves the transformation of the superoxide anion (O_2_•−) into H_2_O_2,_ preventing the formation of the hydroxyl radical (•OH). Subsequently, a combination of enzymes such as peroxidase (POX), catalase (CAT), ascorbate peroxidase (APX), and glutathione peroxidase (GPX) can convert the generated H_2_O_2_ into H_2_O. In addition, glutathione S-transferase (GST) and polyphenol oxidase (PPO) are also implicated in ROS detoxification. However, the ascorbate-glutathione cycle (ASC-GSH cycle) is the predominant antioxidant defense pathway responsible for H_2_O_2_ detoxification. This cycle is composed by nonenzymatic antioxidants, such as ascorbate acid (ASC) and glutathione (GSH), as well as four important enzymes: ascorbate peroxidase (APX), monodehydroascorbate reductase (MDHAR), dehydroascorbate reductase (DHAR), and glutathione reductase (GR) [14].

In the case of PPV-susceptible plants, the infection produces an alteration in both the enzymatic and nonenzymatic antioxidant systems, mainly in chloroplasts. Nevertheless, other compartments, such as symplastic and apoplastic compartments, are also affected. The propagation of the infection causes an imbalance in the antioxidant metabolism, which leads to oxidative stress [9,10,11,15,16]. The observed differences in the leaf antioxidant system between the studies were primarily related to the sensitivity of the *Prunus* species/cultivar to PPV.

In peaches, the symplastic compartment was affected by PPV infection, which produced a significant increase in POX, NADH-POX, and PPO activities [16]; in CAT [10]; and in class I peroxidase (class I APX) [9,17]. In contrast, a significant decrease in SOD activity was reported by Hernandez et al. [9,17]. In apricots, PPV infection produced a drop in class I APX, CAT, POX, and DHAR, as well as an increase in PPO [12,15,16]. 

In the peach apoplastic compartment, PPV induced an increase in class I APX and POX [17] and in NADH-POX, and PPO activities [16,17]. In apricots, the infection produced a decrease in CAT, POX, and SOD activities and a strong increase in PPO [16,17].

In peaches, the chloroplast presented an increase in both class III APX and class I APX activities [9,17], where only the APX enzyme was capable of scavenging H_2_O_2_ in the chloroplast [14]. In contrast, significant decreases in SOD, MDHAR, and GR activities were measured by Hernandez et al. [9,17]. The increase in APX activity could be a delayed stress response and would thus not be capable of avoiding the accumulation of H_2_O_2_. In addition, the decrease in SOD activity could promote ROS accumulation. In apricots, PPV infection produced a decrease in the four enzymes of the ASC-GSH cycle. Concerning SOD activity, the results revealed an increase, which is opposite to what was observed in peaches [12,17]. The altered ASC-GSH cycle, which contributes significantly to H_2_O_2_ removal, could be responsible for H_2_O_2_ accumulation. These alterations may promote PPV-induced oxidative stress in peach and apricot chloroplasts. 

Several studies have investigated the enzymes found throughout the leaves in peach plants and revealed an increase in POX activity [15], similar to the findings previously described in the apoplastic and symplastic compartments of PPV-infected GF-305 plants. The infection also produced a significant increase in GSH and GSSG (antioxidative metabolism); however, PPV had no significant effect on GSH-dependent enzymes [10]. In apricots, an inoculation with PPV resulted in a decrease in SOD, CAT, and GR, whereas a rise in APX, MDHAR, and DHAR activities was observed [18]. These authors demonstrated that the main enzymes of the ASC-GSH cycle were enhanced, while, in other studies [12,15,16], the authors described a decrease in different subcellular compartments.

**Figure 1:**
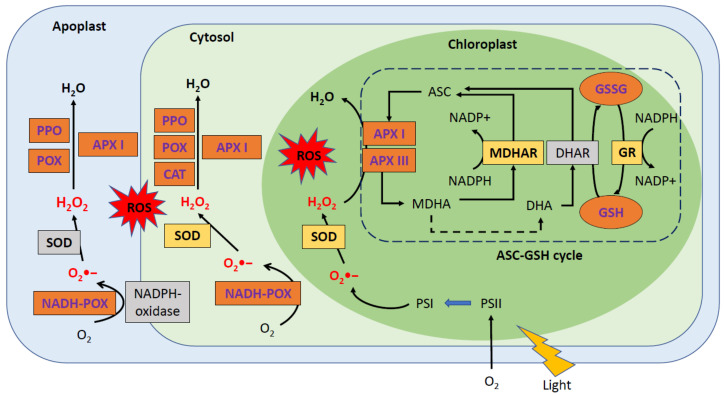
The antioxidant system in peach (GF-305) leaves: main modifications induced by PPV infection. In rectangular form, the enzymatic system, and in oval form, the nonenzymatic system. The orange color indicates an increase of the antioxidant activity, and a yellow color indicates a decrease in the antioxidant activity. The grey color indicates no changes in the enzyme activity. ROS accumulation is shown in red. PPO: Polyphenol oxidase; APX I: class I Ascorbate peroxidase; APX III: class III Ascorbate peroxidase; SOD: Superoxide dismutase; POX: Peroxidase; CAT: Catalase; MDHAR: monodehydroascorbate reductase; DHAR: dehydroascorbate reductase; GR: glutathione reductase; GSH: glutathione; GSSG: glutathione disulfide; ASC-GSH cycle: ascorbate-glutathione cycle; PSI: Photosystem I; PSII: Photosystem II; ROS: Reactive Oxygen Species; H_2_O_2_: Hydrogen peroxide; O_2_•−: superoxide anion.

In summary, PPV infection produced an accumulation of H_2_O_2_ in the symplastic, apoplastic and chloroplastic compartments, despite an overall decrease in SOD activity in peaches [9,15,16]. In addition, a decline in SOD activity suggests a decreased capacity to eliminate O_2_-, promoting ROS accumulation. Several authors have observed that H_2_O_2_-scavenging enzymes, such as induced APX and POX, seem to have failed to regulate their H_2_O_2_ levels and did not avoid damage to the membranes and the development of symptoms resulting from PPV infection [9,16]. Furthermore, the ASC-GSH cycle, the major antioxidant defense pathway to scavenge H_2_O_2_, was also altered by the PPV infection. Below, Figure 1 presents a summary of the major changes induced by PPV infection in peaches (GF-305).

### 2.3. Imbalance at the Phytohormonal Level

PPV infection also caused an imbalance in the phytohormonal profile of susceptible peaches (GF-305). According to Dehkordi et al. [19], the PPV infection could alter their growth, impacting the cytokinin trans-zeatin (tZ) and the gibberellins GA_3_ and GA_4_. In addition, the infection also triggered some stress-related phytohormones, such as the ethylene acid precursor 1-aminocyclopropane-1-carboxylic acid (ACC), abscisic acid (ABA), jasmonic acid (JA), and salicylic acid (SA). In this study, the PPV infection increased the GA_3_ and ABA, while tZ, GA4, ACC, JA, and SA decreased.

SA is involved in several defense mechanisms, such as the establishment of local and systemic resistance, the regulation of small interfering RNAs (siRNAs), the activation of a series of regulatory proteins, such as the non-expressor of Pathogenesis-related genes 1 (NPR1), and transcription factors that regulate defense gene expression, such as the genes of pathogenesis-related (PR) proteins. The SA decrease could induce a susceptibility to PPV infection, despite the presence of other defense responses [19]. For cytokinins, these play an important function in resistance against biotrophs. This anti-biotrophic effect has been described as SA-dependent. Therefore, the reduction in SA signaling could be the cause of the decrease in tZ. Further, JA also contributes to the antiviral response, such as Potato virus Y (PVY) [19]. In the case of PPV infection, the decrease in JA could also promote the infection. Finally, in the advanced stages of viral infection, high concentrations of ABA may induce suppression of the SA and JA signaling pathways, reducing the responses to infection mediated by these phytohormones [19]. In short, a significant increase in ABA in infected leaves is also involved in the susceptibility of peaches.

### 2.4. Defense Responses in Planta at the Transcriptomic Level in Susceptible Varieties

In addition to phytohormonal imbalance, the transcriptome was also altered after virus infection. Transcriptome analysis is essential for the interpretation of the functional elements of the genome and for understanding the cell, organisms, and disease development [20]. Transcriptomic studies were carried out in susceptible peaches (GF-305 and BabyGold 5′) [20,21,22] and apricots (Z506-7 and Canino) [23,24].

**Figure 2:**
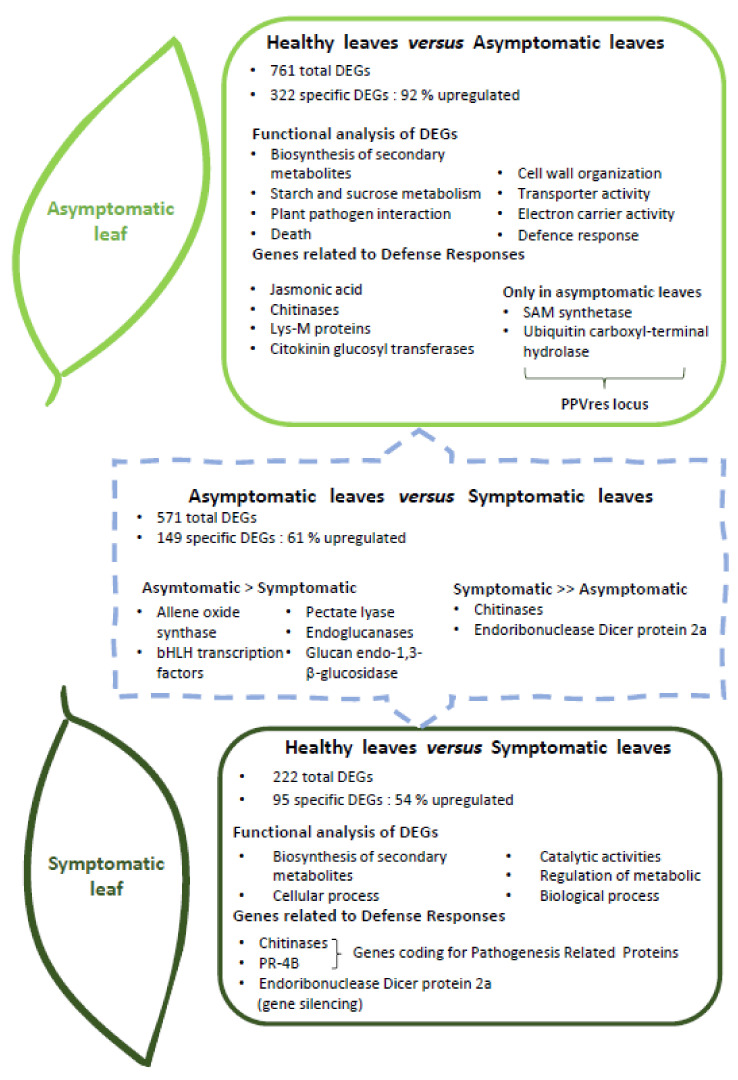
Principal modifications at transcriptomic level after PPV infection in both types of infected leaves: symptomatic and asymptomatic. The information shown in this figure is based on a study by Rubio et al. [20]. DEGs: differentially expressed genes.

Rubio et al. [20] were the first authors to compare healthy, asymptomatic, and symptomatic leaves during PPV infection in peaches (GF-305). This study provided a global picture of the peach transcriptome after PPV infection and the development of sharka symptoms. In fact, transcriptome modifications were induced by the PPV infection but differ according to the type of tissue, with or without symptoms. Asymptomatic leaves presented a greater number of deregulated genes as a result of PPV infection than symptomatic leaves. Furthermore, 92% of specific differentially expressed genes (DEGs) were upregulated in asymptomatic leaves, while only 54% were upregulated in symptomatic leaves. The different trends between symptomatic and asymptomatic leaves are summarized in Figure 2.

In addition, the authors performed a functional analysis of DEGs in symptomatic and non-symptomatic peach leaves based on a gene ontology (GO) enrichment analysis and KEGG orthology (KO) assignments. Regarding KO assignments, Rubio et al. [20] reported that the “biosynthesis of secondary metabolites” was the most represented functional annotations for both symptomatic and asymptomatic peach leaves. Nevertheless, “starch and sucrose metabolisms” and “plant–pathogen interactions” were only represented in asymptomatic leaves when compared to control leaves, suggesting a defense response that prevents virus accumulation. For the GO enrichment analysis, the secondary GO term “Cellular Process” was underrepresented, whilst the genes implicated in “catalytic activities” and “regulation of metabolic” and “biological processes” had high representation in the symptomatic leaves. In the case of infected yet asymptomatic peach leaves, genes belonging to “death”, “cell wall organization”, “transporter activity”, and “electron carrier activity” were more abundant. A summary of the main annotations proposed by Rubio et al. [20] are presented in Figure 2. In addition, Zuriaga et al. [24] demonstrated, in a susceptible apricot variety named “Canino”, that several GO were significantly enriched, concerning molecular functions (binding and catalytic activities); biological process (abiotic stimulus response); and cellular component (related to the chloroplast, nucleus, and nucleosome).

Concerning defense responses, Rubio et al. [20] reported that genes encoding the two pathogenesis-related proteins (*chitinase* and *PR-*4B) were overexpressed in symptomatic peach leaves. Additionally, *endoribonuclease Dicer protein 2a*, involved in “metabolic processes”, was significantly upregulated in symptomatic peach [20] and apricot [23] leaves. This protein is implicated in antiviral defense through RNA silencing, and its overexpression in leaves with strong symptoms may indicate the suppression of a gene-silencing mechanism in the plant by PPV HCPro and P1 PPV proteins. Rubio et al. [23] analyzed the susceptible apricot “Z506-7” under PPV infection through a comparison of healthy and infected leaves that exhibited sharka symptoms. In general, symptomatic apricot leaves presented similar results to peach. In this study, DEGs were mainly associated with the response to different stimuli, such as JA, SA, wounding, herbivore attack, biotic stimulus, and anthocyanin and proanthocyanin biosynthesis processes. Wang et al. [22] also suggested that genes involved in defense, cellular transport, development, protein synthesis, and proteins with binding functions were induced after PPV infection in peaches (BabyGold 5′). For instance, some genes were highly prevalent in symptomatic leaves, including B-1,3-glucanase, cytochrome-P450-like protein, cytochrome P450 monooxygenase, heat-shock protein 70, thioredoxin, alcohol dehydrogenase, catalase, cysteine protease inhibitor, translation factor EF-1a, and pathogenesis-related protein (PR1). The translation factor EF-1a is a component of the protein complex that could act as a host factor that assists in the translation of viral proteins. In contrast, heat-shock protein 70 is related to the induced resistance response of the host plant, and the genes of the PR protein are general stress-responsive genes. Curiously, Clemente-Moreno et al. [21] reported that PPV infection induced *NPR1* in peach leaves. NPR1 is a key regulator in the signal transduction pathway that leads to systemic acquired resistance (SAR) and is implicated in the activation of PR gene expression. NPR1 induction could be activated by GSH and H_2_O_2_, both compounds induced by PPV infection [21].

Rubio et al. [20] also identified several DEGs in asymptomatic leaves. These genes included several disease resistance proteins, such as chitinases and *Lys-M* proteins, proteins involved in jasmonate (JA) biosynthesis, such as *allene oxide synthase* and *allene oxide cyclase 4 chloroplastic*, and signaling, such as *S-adenosyl-methionine* (SAM) *synthetase 2* and a *bHLH transcription factor*. In addition, JA-responsive genes involved in defense, as well as jasmonate and some *cytokinin glucosyl transferases*, were induced. Moreover, higher patterns of expression were observed in genes related to the defense response and the response to biotic stimuli, including *pectate lyase*, *endoglucanases*, and *glucan endo-1,3-b-glucosidase.* Furthermore, SAM synthetase and *ubiquitin carboxyl-terminal hydrolase* were only overexpressed in the non-symptomatic leaves. Both genes are localized in the PPV-resistant locus (*PPVres*). The *PPVres* locus contains predicted transcripts that were previously reported to confer resistance against potyviruses, such as SAM. Finally, *4-coumarate-Coa ligase-like 9* showed the same trend as SAMs. DEGs implicated in the defense responses in symptomatic and asymptomatic leaves described by Rubio et al. [20] are illustrated in Figure 2.

The modifications differed between symptomatic and asymptomatic leaves. While the number of DEGs and secondary GO terms were greater for asymptomatic leaves, the differences from the background were more significant for symptomatic leaves. The functional enrichment analyses were also consistent with the hypothesis that biotic stress marks a transition from growth and reproduction to a physiology and metabolism tailored to defense responses [23]. In symptomatic peach and apricot leaves, the defense responses suggest that susceptibility to PPV is a complex process based on a continuous battle between the PPV and the plant at the pathogen resistance gene level, including gene silencing. Conversely, the specific defense responses that occurred in asymptomatic leaves, such as SAM synthetase, could suggest the existence of an active reaction, which limits the appearance of symptoms throughout the whole *Prunus* tree [20].

### 2.5. Alterations at the Proteomic Level

PPV infection also produced alterations in the proteome from susceptible peach GF-305 [11]. Diaz-Vivancos et al. [16] found that PPV infection induced an increase in thaumatin-like proteins, as well as a decrease in mandelonitrile lyase (MDL) in apoplasts. MDL is a flavoprotein involved in the catabolism of (R)-amygdaline. The thaumatin-like protein is a PR protein involved in plant protection. The induction of this protein could be mediated by an increase in H_2_O_2_ after PPV infection. Furthermore, the increase in thaumatin-like proteins was confirmed in symptomatic peach leaves by a transcriptomic analysis [20]. In addition, Clemente-Moreno et al. [11] described the differential protein expression in PPV-infected peach plants. PPV infection reduced the abundance of proteins related to photosynthesis, such as ferredoxin-NADP(H) oxidoreductase; to carbohydrate metabolism, such as ADP-glucose pyrophosphorylase; to amino acid metabolism; and to photorespiration. As explained by Clemente-Moreno et al. [10], ferredoxin-NADP(H) oxidoreductase is involved in the electron transport chain, and therefore, PPV infection could reduce photosynthesis and induce ROS accumulation. Moreover, the decrease in the ADP-glucose pyrophosphorylase combined with the decline in the photosynthesis rate are possible sources for the decline in starch in peach leaves. In contrast, other polypeptides related to photosynthesis increased after PPV infection. The infection also induced the expression of proteins related to the stress response, such as benzoquinone reductase and a putative chaperone clpb [10].

### 2.6. Translatomic Analysis: An Original Approach to Study the Impact of PPV Infection

To go even further, Collum et al. [25] analyzed the translatome profile of European plum leaves after PPV infection. The aim was to understand the responses to PPV infection in phloem and non-phloem tissues during leaf development. The translatome refers to all mRNAs associated with ribosomes for protein synthesis by translation. The analysis of the translatome is critical for understanding gene expression and can reveal important regulatory cues and relevant pathways linked to disease [26]. Therefore, an investigation of the phloem translatome is of interest for the study of host–pathogen interactions. Phloem plays a fundamental role in plants by enabling the long-distance transport of hormones, nutrients, proteins, RNAs, and carbohydrates that are essential for plant growth and development, as well as allowing the plant to respond to a diverse array of abiotic and biotic stresses. Phloem also allows plant pathogens to spread systemically throughout a host plant [27]. For instance, PPV can move long distances through the phloem vascular system. Viral proteins such as VPg, 6K2, and CP are required for the long-distance movement of PPV through phloem, while some host factors such as the RTM genes restrict the long-distance movement of PPV [25].

In this study, Collum et al. [25] aimed to identify the specific responses of phloem and non-phloem tissues. For this purpose, the authors collected samples covering the secondary morphogenesis at 2-, 4-, 6-, and 12-weeks post-cold-induced dormancy. The authors used the translating ribosome affinity purification (TRAP), followed by RNA sequencing; this methodology was explained by Collum et al. [25,27].

**Figure 3:**
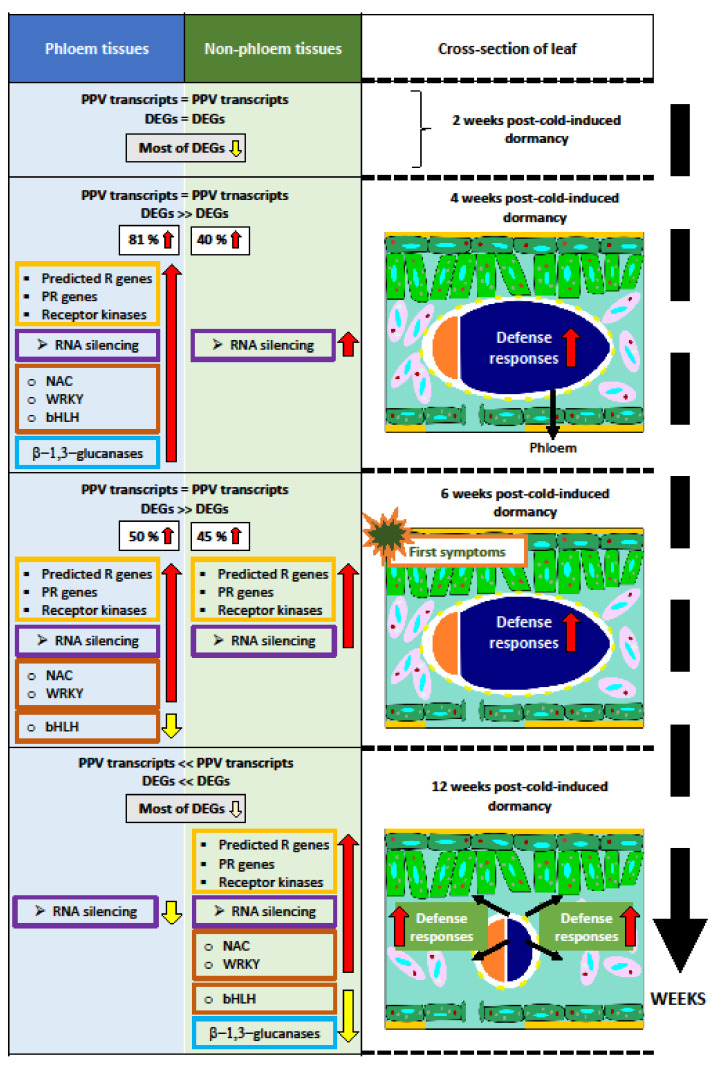
Temporal and spatial dynamics of leaf tissues responses at the translation level to virus infection. The figure is separated into three parts: in blue (left), the modifications induced in the phloem tissues; in green (middle), the modifications induced in the non-phloem tissues; and on the right, a cross-section of a leaf illustrating the main modifications. Red arrows indicate overexpression, and yellow arrows indicate under-expression. Differentially expressed genes (DEGs) have been grouped by color for a better overview. The major responses to PPV infection are expressed early in phloem tissues and are manifest later in the non-phloem tissues. Results correspond to Collum et al. [25]. bHLH: basic-helix-loop-helix and NAC transcription factor: NAM, AFAT, and CUC.

In general, the total number of DEGs increased in infected leaves during leaf development. During the first six weeks, the levels of viral transcripts were similar for both phloem and non-phloem tissues. In addition, the first symptoms appeared from week 6 post-cold-induced dormancy. At 2 weeks, both tissues presented an equal number of DEGs, and most were downregulated. Nevertheless, at 4 and 6 weeks, the phloem was highly responsive to PPV infection. At 4 weeks, DEGs were mostly upregulated in phloem, with an increase of 81%, compared to 40% for non-phloem. By 6 weeks, the upregulation was a more equal split, with 50% of DEGs for phloem tissues and 45% for non-phloem tissues. In contrast, in mature leaves (12 weeks), the number of DEGs significantly decreased in phloem tissues but remained constant in non-phloem tissues. These modifications were correlated with a significant decline in PPV transcripts only in phloem tissues. In addition, most DEGs were downregulated again in both types of tissues. The number of DEGs and their overexpression were significantly higher and occurred earlier in phloem tissues, revealing that phloem tissues are highly responsive to PPV infection during the first stages. These different behaviors between the phloem and non-phloem tissues are presented in Figure 3.

Several categories of genes were affected by PPV infection, but they were primarily involved in the stress response and were upregulated in both the phloem and non-phloem tissues. Nevertheless, in phloem tissues, these defense responses were triggered earlier than in non-phloem tissues, mainly at 4 weeks. In later weeks, at 6–12 weeks, the defense responses were significantly enriched in non-phloem tissues. These defense responses included genes related to predicted R genes with NBS-LRR domains, PR genes, and receptor kinases. Some genes, such as Toll interleukin 1 receptor-NBS-LRR (TIR-NBS-LRR) and coiled coil-NBS-LRR (CC-NBS-LRR) were exclusively upregulated in phloem tissues, indicating a specificity of these genes in phloem tissues during virus infection. Furthermore, predicted homologs of PR genes PR1 and PR5 showed the same pattern, with an early increase in phloem tissues (week 4) and a delayed increase in non-phloem tissues (week 12). PR1 may be associated to SA-mediated defense responses, which have an impact in PPV systemic movement. In addition, genes associated with RNA silencing such as dicer-like (DCL) endonucleases, RNA-dependent RNA polymerases (RDR), siRNA methyltransferase (HEN), and argonaute proteins (AGO) were also affected during PPV infection. From week 4 to week 6, activation of these genes was generally constant in both phloem and non-phloem tissues. Nevertheless, at 12 weeks, the levels of the induced silencing genes significantly reduced only in phloem tissues, in correlation with the reduction of viral transcripts. The activation of silencing-associated defenses correlated with the presence of the virus in the tissue type.

Interestingly, the highest levels of silencing activity occurred in phloem tissues, particularly in companion and bundle sheath cells. This suggests that these cells are very sensitive to the activation of silencing defenses, promoting the rapid transport of silencing both locally and systemically. Concerning the defense-associated transcription factors, four families, including NAC (NAM, AFAT, and CUC); WRKY; bHLH (basic-helix-loop-helix); and MYB (myeloblastosis), showed some level of upregulation in phloem tissues at 4 weeks. The NAC and WRKY genes appeared to be maintained and upregulated in phloem tissues at 6 weeks, whereas, in non-phloem tissues, both families were upregulated only at week 12. For example, the WRKY50 genes were upregulated only in phloem at 4 weeks. WRKY50 activated the expression of PR1, which was also upregulated at four weeks in phloem tissues. Conversely, the bHLH genes were predominantly downregulated in phloem tissues at 6 weeks and later in non-phloem tissues (week 12). These transcription factors are involved in the JA signaling pathway, which acts antagonistically to SA-mediated pathways. The general downregulation of the bHLH genes and the upregulation of the WRKY and NAC genes at twelve weeks would potentially reinforce the activation of SA-associated defense mechanisms. At 12 weeks, genes related to cell wall modification, β-1,3-glucanases, and auxin-responsive genes were primarily downregulated in non-phloem tissues during PPV infection. β-1,3-glucanases are involved in the degradation of callose, which can block viral movement. Thus, their downregulation could be effective in stopping the spread of the virus. However, some β-1,3-glucanases were upregulated in four-week-old phloem tissues, which could reduce the callose deposition in the phloem plasmodesmata. These host responses in non-phloem tissues occurred too late and could not prevent the systemic spread of the virus. Some of these defense responses are illustrated in Figure 3, reflecting the temporal and spatial dynamics of leaf tissue responses to infection and the premature response of phloem tissues.

In conclusion, Collum et al. [25] described the alterations caused by the PPV infection and revealed that these changes were dynamic. In the early stages, phloem tissues displayed a significantly higher level of response to PPV infection than non-phloem tissues, regardless of the similar levels of the viral transcripts. In contrast, in mature 12-week-old leaves, the non-phloem tissues became the predominate altered tissue. Interestingly, the increase in plant defense responses was related to the presence of active viral infection, particularly induced in phloem tissues between weeks 4 and 6. The PPV infection induced a temporal and spatial coordination of the defense responses in phloem and non-phloem tissues and revealed the importance of phloem responses within a perennial host.

## 3. How Does the *Plum Pox Virus* Affect the Resistant *Prunus* Leaves?

Plants have evolved in environments rich in sources of stress, whether biotic or abiotic. When it comes to viruses, there is a wide variety, with a number of methods of transmission or infection. To fight against this source of stress, several defense mechanisms have been established by plants. These mechanisms are designed to protect the body against infection by containing or eliminating it. Conversely, viruses have evolved in such a way as to override these defense mechanisms and to still be able to develop in their host. This course in evolution has yielded a great diversity of countermeasures and defenses by plants and pathogens. Thus, different responses can be observed following the same source of infection, even for plants of the same species. In this section, we will review the various studies that describe the responses observed at the transcriptomic or metabolic levels following infection by PPV in species of the genus *Prunus*.

### 3.1. Major Changes in the Antioxidant System as Result of PPV Infection in Resistant Varities

Cells naturally produce antioxidant substances to protect themselves from oxidative stress, which can lead to cell destruction. During a PPV infection, the antioxidative system is profoundly altered, leading to an increase in ROS.

Few studies have been conducted on the oxidative stress associated with PPV infection. The first study was published by Hernandez et al. [18]. Comparisons between susceptible (Real Fino) and resistant (Goldrich) apricot cultivars were made on different antioxidant enzymes. The results showed a modification in enzyme activities between the two varieties. The resistant variety showed an increase in enzymatic activities related to the antioxidant system of the cells.

**Figure 4:**
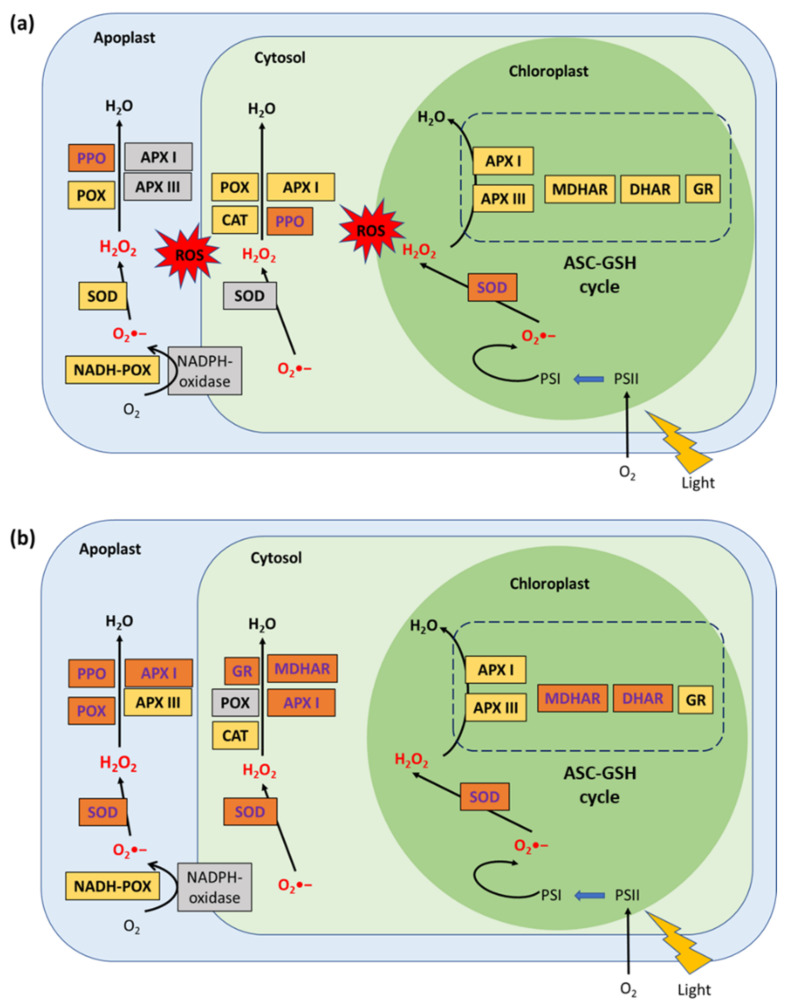
Differences in the antioxidant system between susceptible and resistant apricot cultivars. (**a**) Changes in the antioxidant system in susceptible apricot cultivars. (**b**) Changes in the antioxidant system in resistant apricot cultivars. The orange color indicates an increase of the enzymatic activity, and the yellow color indicates a decrease in the enzyme activity. The grey color indicates no changes in the enzyme activity. PPO: Polyphenol oxidase; APX I: class I Ascorbate peroxidase; APX III: class III Ascorbate peroxidase; SOD: Superoxide dismutase; POX: Peroxidase; CAT: Catalase; MDHAR: monodehydroascorbate reductase; DHAR: dehydroascorbate reductase; GR: glutathione reductase; GSH: glutathione; GSSG: glutathione disulfide; ASC-GSH cycle: ascorbate-glutathione cycle; PSI: Photosystem I; PSII: Photosystem II; ROS: Reactive Oxygen Species; H_2_O_2_: Hydrogen peroxide; O_2_•−: superoxide anion.

In another study, Diaz-Vivancos et al. [16] studied the enzymatic activities in different cell compartments. Thus, differences in expression were observed in contaminated plants, including superoxide dismutase (SOD) activity (antioxidant enzyme), which increased in resistant varieties but decreased in susceptible varieties in apoplasts and cytosols. In addition, a difference in activity in ascorbate peroxidase (APX) (an oxidoreductase) in the apoplast space was also observed. In chloroplasts, resistant varieties showed an increase in activity of the enzymes DHAR and MDHAR. Both enzymes are linked to the ascorbate-glutathione cycle (ASC-GSH cycle), whose role is to eliminate the H_2_O_2_ present in the chloroplast. Thus, a link between H_2_O_2_ and the response to PPV was proposed by the authors in virus-resistant varieties. The roles of H_2_O_2_ have since been widely described. Although, at high doses, this molecule is toxic, at low doses, it acts as a signal to allow the modification of cellular responses in case of stress. Hernandez et al. [17] published a paper that highlighted the different modifications in the different cell compartments of plant cells following PPV infection. A comparison between susceptible and resistant apricot varieties is shown in Figure 4.

### 3.2. Phytohormonal Level: Transfer of Resistance to PPV by Grafting “Garrigues” Almonds to Peaches

The main function of phytohormones is to transmit a signal within the body or to plants in proximity, especially when the plant is under stress. For example, salicylic acid is an aromatic compound produced by plant cells involved in numerous types of stress. In the event of infection by a pathogen, infected cells synthesize SA via secondary metabolite pathways and then transport it to healthy cells. This not only builds up acquired local resistance but also transmits a signal to the whole plant, thus triggering systemic resistance.

Several studies have been conducted to determine the impact of SA in the defense against PPV. Bernal-Vicente et al. [28] carried out a study on the peach variety GF-305 in order to identify a third synthetic pathway based on mandelonitrile (MD). Plants infected with PPV were treated with MD and phenylalanine (Phe). The results showed that not only did the SA content increase with MD treatment but there was also an increase in ABA and JA. These plant hormones are known to be linked to stress in plants. In addition, MD and Phe treatments reduced the PPV-related symptoms, although Phe had a lesser effect than MD. We can therefore assume that the increase in SA in plants reduces the symptoms associated with PPV [28]. In addition, researchers hypothesized that ABA might serve as a regulator of the plant defense response in *Arabidopsis thaliana* and cucumbers following infection with cucumber mosaic viruses, two viruses from the same family as PPV. Therefore, the increased levels of both SA and ABA are thought to be a plant defense response to PPV infection [29].

**Figure 5:**
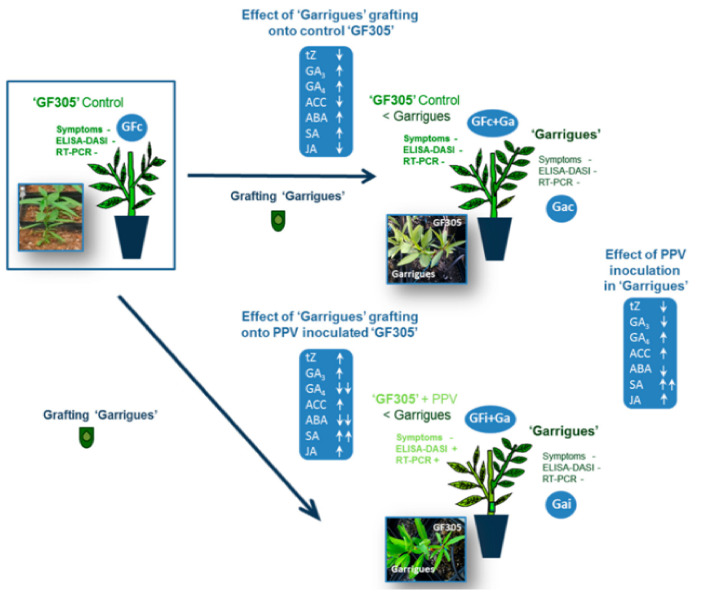
Summary of the differences observed in the production of hormones according to the different conditions (Control GF-305, Control GF-305 grafted with Garrigues, Infected GF-305 grafted with Garrigues, and Garrigues-infected). tZ: Cytokinin trans-zeatin; GA3 and GA4: Gibberelines; ACC: ethylene acid precursor 1-aminocyclopropane-1-carboxylic acid; SA: Salicylic acid; JA: Jasmonic acid; ABA: Abscisic acid. Adapted from Dehkordi et al. [19].

In another study, Dehkordi et al. [19] compared the hormone level profiles between susceptible (GF-305), resistant (Garrigues), and Garrigues grafted onto GF-305 under healthy and infected conditions. Grafting Garrigues on GF-305 not only altered the hormonal profile in healthy GF-305 plants but also reduced the symptoms and prevented the onset of necrosis or a hypersensitive response (HR) in infected GF-305 plants. As previously described, GF-305 plants experienced a drastic drop in SA levels after infection with the virus, while, in the controls, grafting with Garrigues increased their levels. Another hormone, JA, also appears to be very important in the defense against mosaic viruses. A decrease in JA seems to favor the accumulation of PVY in the early stages of the infection. In addition, an increase in JA also helps to promote plant defense against PVY and *Potato virus X*, although this increase does not occur in the early stages of infection. Since this virus is a Potyvirus, one might think that JA is also very important in the tree’s defense against PPV. This response induced by the resistant rootstock has been described as an acquired systemic response (SAR). In addition, this SAR transmission was mediated through xylem vessels, altering the contents of the antioxidant enzymes and the presence of molecules such as SA [19]. Figure 5 summarizes the results of the study and allows the differences in the hormonal profiles of the plants to be grouped together under different conditions.

### 3.3. Defense Responses in Planta at the Transcriptomic Level in Resistant Varities

#### 3.3.1. Overview: Transcriptome Modifications in Resistant Plants

The study of the transcriptome makes it possible to determine which genes are involved in the various response mechanisms and may make it possible to determine which pathways are used next. Rubio et al. [23] studied the difference in gene expression between two varieties of apricot: “Rojo pasion”, a resistant variety (Control named Rc and infected named Ri), and Z506-7, a susceptible variety (Control named Zc and infected noted Zi). An RNA-Seq analysis revealed the existence of more than 2005 DEGs. The comparisons were as follows: Rc versus Ri (164 DEGs), Zc versus Zi (239 DEGs), Rc versus Zc (803 DEGs), and finally, Ri versus Zi (302 DEGs). In another study, Rubio et al. [20] demonstrated a difference in expression of 15,544 genes after infection of the peach variety GF-305. Analyses on apricots demonstrated that many genes were over-represented in different pathways, like the metabolism of terpenoids and polyketides, flavonoid and anthocyanin biosynthesis, porphyrin, or chlorophyll. The study of the difference in gene expression between varieties showed that some genes were over-represented. Proteins from these genes are, for example, linked to terpenoids (hydrocarbons possessing additional functional groups, which are found in many species of plants); polyketides (a family of secondary metabolites possessing great pharmacological capacities (antibiotics, antimicrobials, antifungals, etc.); porphyrin (a family of molecules including chlorophyll); chlorophyll; and fatty acids, as well as the biosynthetic pathway of flavonoids (secondary metabolites found in all vascular plants used in defense against pathogens) and anthocyanin. It is noted that many pathways that are overexpressed in resistant plants during infection with PPV are used in the defense of pathogens or in response to stress. It is therefore likely that these metabolites play an important role in the plant’s defense against the virus.

Moreover, other genes have been identified as linked to resistance or susceptibility to PPV, including endoribonuclease Dicer homolog 2a (Dicer 2a) or the Muscle LIM protein-like protein 423 (MPL). Comparisons between susceptible and resistant varieties revealed an under-expression in resistant varieties for Dicer 2a in both healthy and infected conditions. As previously described, this protein is involved in the gene silencing mechanism and could be inhibited by the HCPro and P1 PPV proteins. It appears that resistant varieties succeed in repressing the HCPro and P1 viral proteins [23].

With regards to the MLP-like protein, it is over-represented in the susceptible genotype when comparing Zc versus Rc and Zi versus Ri. This gene encodes a protein associated with a stress response or in phytohormonal signaling. Finally, gene coding allene oxide synthase is also more expressed in the susceptible genotype during comparisons made between the controls (Zc versus Rc). Its main role is to catalyze the first step in the biosynthesis of JA.

#### 3.3.2. Regulation of Genes Coding for MATH Domain in PPVres Locus

In apricots (*Prunus armeniaca*), a resistance locus of around 196 Kb protects several varieties. This locus is present in the majority of varieties that possess a disease resistant trait. Zuriaga et al. [24] enabled the characterization of a transcriptomic response to infection with the PPV-D strain by comparing the responses of two resistant varieties of apricot (“Goldrich”, a heterozygote resistant/susceptible variety, and “Stella”, a homozygote resistance) to a susceptible one, “Canino”. The PPV infection altered the *ParP-1*–*6* genes, which are located in the locus *PPVres*. It was observed that genes ParP-3 and ParP4 are downregulated in the “Stella” cultivar in comparison to the susceptible varieties.

The differential expression of these genes was confirmed by q-RT-PCR. No difference was observed between the healthy and contaminated groups for the “Stella” cultivar. It is therefore not a response to infection on the part of the host but seems to be an innate resistance.

The authors hypothesized that *ParP 3* and *ParP 4* are implicated in PPV susceptibility, and that *ParP 4* downregulates the expression of its functional paralog *ParP 3* by silencing. Therefore, their inhibition would confer host resistance to PPV. These genes are therefore good candidates for the improvement of varieties though the selection of markers.

#### 3.3.3. eiFiso4G Transcription Initiation Factor: A Key Role in Virus Replication

Initiation factor (IF) is a protein that helps the ribosome initiate the translation of mRNAs. Many pathogen susceptibility genes have been described as members of initiation complexes, such as eIF4E. Rubio et. [30] attempted to determine the role of transcriptional initiation factors in the PPV infection of Japanese plums (*Prunus salicina*). In fact, a mutation in these genes confers a resistance to infection by viruses from the *Tombusviridae, Bromoviridae, Waikaviridae*, and *Potyviridae* families. A previous study demonstrated that the eIFiso4E factor played an essential role in the infection of *Arabidopsis thaliana* by PPV [31]. To determine the role played by this translation initiation factor, silencing constructions were carried out on *Prunus salicina* plants for the orthologous factors 4E and 4G of *Prunus persica*. This silencing was performed using interfering RNA constructs. Subsequently, regeneration of the plants was carried out in the same way. The transgenic lines were grafted onto PPV sensitive rootstocks.

**Figure 6:**
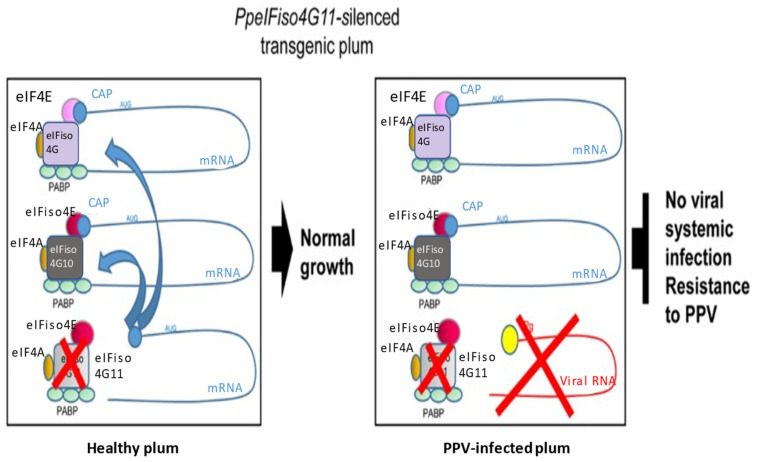
Model of eIFiso4F-mediated susceptibility to PPV in diploid plums from Rubio et al. [30]. The silencing of eIFiso4G, a translation initiation complex, prevents the development of the virus in the host but does not prevent the development of the plant. Translation initiation factors: eiF4E, eIFiso4E, eIFiso4G, eIFiso4G11, eIFiso4G10, and eIFiso4A; PABP: poly(A)-binding protein; CAP: catabolite activator protein.

Two loci on the *eIFiso4E* gene (named *PpeIFiso4G10* and *PpeIFiso4G11*) were detected by a BLAST analysis. A reduction in the expression of PpeIFiso4G11 allowed one of the transgenic plants to resist infection by PPV-M and PPV-D. In addition, this resistance could be transmitted to grafts of the plant, which seems to indicate that this resistance is stable and durable. In addition, the decrease in expression of one of the genes encoding for the initiation factor eIFiso4G is linked to the production of specific 21–24-nt siRNAs, interfering with the production of *PpeIFiso4G11*. An infection model was thus created in which the PPV hijacks the eIFiso4F factor. If the transcription factor is not present, the PPV will not be able to use the transcription factor to replicate itself. However, the cell will still be able to recruit other transcription factors to continue normal cellular activities (see Figure 6).

In another study, Wang et al. [32] also succeeded in conferring resistance to PPV in plum plants. For this, the authors silenced the transcription factor eIF4E and its isomer, eIF(iso)4E. The latter must bind to the transcription factor eIFiso4G11 in order to recruit ribosomes to initiate mRNA translation.

### 3.4. Case Study: ‘Jojo’ Variety, An Example of the Hypersensitive Response

The purpose of HR is to prevent a disease from spreading to the rest of the plant. This response is characterized by the rapid death of infected cells. Among the different *Prunus* species, some are known to produce an HR in response to PPV infection. The variety “Jojo” is a good example. Rodamilans et al. [33] compared the transcriptomic profile of this variety before and after infection in order to detect the mechanisms linked to this response against the PPV-D strain. A number of (2234) genes were significantly upregulated and 786 were downregulated in infected plants.

The overexpressed genes were associated with the cell wall, the cytoskeleton, or the chromosome (cyclin-like proteins). Conversely, under-expressed genes were linked to the transport of electrons from photosynthesis or to the thylakoids. For all genes, 1094 were linked to Pathogen Receptor Genes (PRGs), proteins that play a key role in the recognition of pathogen avirulence genes. Among these genes, 154 were differentially expressed by PPV infection (126 overexpressed and 28 under-expressed). Half of them corresponded to genes suspected of having kinase activity, and 13% of the overexpressed genes coded for the LRR (Leucine-rich repeat) receptors. These genes are critically important in resistance to viral infections [33]. However, among the 3020 DEGs, many are not described as resistance genes but may be implicated in the mechanism of the HR. After analysis, 75 genes were identified as those with a potential role in the response mechanism to PPV infection. These genes code for different proteins, such as polyphenol oxidases, histones, or aspartic proteases.

In another study, Markiewicz et al. [34] identified 27 genes implicated in HR against PPV infection in the “Jojo” variety. These genes were selected by comparing hypersensitive and susceptible varieties in healthy and PPV-infected conditions. Among these genes, 15 were overexpressed in a hypersensitive variety under PPV infection and seemed to be specific to HR. In addition, 11 genes showed a differential expression between the two varieties (hypersensitive versus susceptible) but were not affected by an infection within the same variety. Only one gene was differentially expressed as a result of PPV infection in the susceptible variety. Most of the identified genes are involved in defense functions in several host–pathogen interactions. The others have different functions, such as transcription and translation, signal transduction, or transport.

In general conclusion, we observed large differences in the PPV response between susceptible and resistant varieties in peaches and apricots. Resistant varieties not only can prevent the virus from hijacking its host’s metabolism for replication but also have wide range of signaling and defense mechanisms, including hormones. It is also interesting to note that transcriptomic analyses revealed that not all the genes altered by PPV infection were involved in the defense mechanisms.

## 4. How Does the *Plum Pox Virus* Affect the Susceptible *Prunus* Fruit?

PPV is one of the most studied plant viruses, yet few studies have focused on the effect of PPV infection in *Prunus.* In addition, most available data in the literature only concerns leaf tissues. Therefore, this part of the review focuses on the modifications induced by PPV infection in *Prunus* fruits. While the main studies concerned plum trees [35,36,37,38], here, we also present relevant studies on apricot trees [39] and peach trees [40].

Many factors, including the virus strain and isolates, tree cultivar and age, rootstock, cultural practices, time and mode of infection, and presence of vector species, as well as climatic conditions, could influence the impact of PPV on fruit quality and production [40]. Unfortunately, parameters between studies are not always the same, and sometimes, opposite results can be explained by the influence of different parameters.

The PPV infection affects *Prunus* fruits at different levels, such as yield and the physical properties of the fruits: primary compounds such as the soluble solids content, sugars, and organic acids and secondary compounds such as phenolic compounds.

### 4.1. Yield Attributes and Physical Properties of Fruit

Many parameters were influenced by PPV infection, including vegetative growth, yield, economic results, physical properties of the fruits, and the ripening process. Milošević et al. [39] studied the influence of a mixed infection of PPV-D and PPV-Rec on susceptible apricot trees that were long-term infected for at least 5 years. Generally, the external symptoms on apricots are not as severe as on plums; on the leaves, the symptoms are not clear, and they are scarce on the skin of the fruits, but on the stones, the symptoms are commonly expressed. As expected, trees that were infected over a long period of time showed mild symptoms on the fruits, but on stones, the symptoms were evident. Symptoms on the leaves were severe [39]. The authors proposed that the results obtained may be explained by the long-term and mixed infections but, above all, by the high virulence of the PPV-Rec strain.

Concerning vegetative growth, the trunk cross-sectional area (TCSA) value was significantly reduced by PPV infection in apricots [39]. In an earlier study, the authors highlighted a significant reduction in vegetative growth in infected susceptible plums [35]. In contrast, Samara et al. [40] noted that, for peaches, no difference in tree growth was observed. However, peach trees were infected by the Ontario PPV-D strain, which is different from the other reported PPV-D isolates. Visually, infected trees were indistinguishable from healthy trees in their overall appearance [40]. PPV could also induce a massive premature drop [35,36,37] and, consequently, an important decrease in total yields per tree and unit area in infected versus noninfected plum and apricot trees [35,39].

The physical properties of the fruits were also modified. In apricots only, the flesh firmness and flesh rate significantly decreased. Data for the firmness indicated that infection causes earlier fruit ripening, because changes in firmness is a useful indicator of stone fruit maturity. A lower flesh rate was reported as a consequence of a nonsignificant decrease in fruit weight and an increase in stone weight [39]. In addition, in some cases, PPV could cause a negative effect on the average fruit weight and fruit dimensions [35,36,37,40]. The colorimeter measurements on the fruit skin were also modified [36,40]. Colors of the infected fruits changed prematurely and reflected both a stress response and advanced ripening [40].

In summary, PPV infection seems to produce an earlier fruit ripening [37,38,41], which may also have an effect on primary compounds such as the soluble solids content, sugars, and organic acids and, consequently, on the fruit quality. These parameters are described below.

### 4.2. Primary Compounds

#### 4.2.1. Soluble Solids Content (SSC)

Soluble Solids Content (SSC) are composed of sugars, organic acids, vitamins, proteins, free amino acids, essential oils, salts, and glucosides [39]. Ripening increases the SSC, an essential quality variable in stone fruits, which has been proposed as the most reliable maturation parameter for stone fruits. Milošević et al. [39] reported that PPV infection induced a premature ripening and significantly higher SSC in apricot fruits from infected trees than in the uninfected trees. Interestingly, this result is not in agreement with previous studies, which reported slightly higher values in apricot fruits from uninfected trees, and no significant differences in peaches [39]. For infected plums, SSC increased prematurely compared to healthy plums [36].

#### 4.2.2. Sugars: Content and Composition

Modifications produced by PPV in the sugar content and composition were analyzed in susceptible plums [36,37] and apricots [39]. In plum studies, the effects of PPV-Rec with different durations of infection were studied during the last 3 weeks of ripening, comparing healthy trees with trees infected over a short term (at least 1 year) and those infected over a long term (at least 5 years). To evaluate the ripening process, fruits were hand-picked from all trees on three sampling dates, at days 0, 9, and 22, and for the healthy trees, fruits were also sampled on day 30. Additionally, Usenik and Marn [37] also observed the accumulation in necrotic tissue from long-term infected fruit.

In contrast to infected trees, premature fruit dropping from healthy trees was minimal, which indicates that fruit could reach the optimal amount of sugars. Premature fruit dropping and symptoms are accentuated with respect to the duration of infection [37]. In general, the relative content of sugars increases during ripening, with an increase in the relative sucrose content, while glucose, fructose, and sorbitol decrease during the final phases of ripening. Despite the decrease of the glucose and fructose levels, the total content of fermentable sugars (FS) increases [36,37].

The fruits from long-term infected trees had less total sugars than the other treatments [36,39]. Conversely, Usenik and Marn [37] reported no significant differences for total sugars, with the exception of a significantly lower content in necrotic tissue on the first two picking dates. Many studies have demonstrated that the composition of FS in long-term infected fruit differs from short-term infected and healthy fruits. It can be assumed that fruits sampled on the first two dates mimic the properties of prematurely dropped fruits, since plum fruits stop producing sugars once removed from their carbon source [37]. On day 0, glucose was the prevailing sugar in all the treatments. This changed in favor of sucrose on day 9 in all the treatments except for necrotic tissue. On day 30, sucrose was the predominant compound in healthy fruits, followed by glucose and fructose. Fruits from long-term infected trees have less glucose than short-term infected and healthy fruits. In the case of sucrose, the increase during ripening was faster in both infected trees, which, consequently, had more sucrose at day 22 [36,37]. For apricots, however, sucrose was higher in uninfected fruits than in long-term infected fruits [39]. In long-term infected fruits, sucrose and glucose were more quickly modified [37]. In addition, the fructose content decreased and showed an opposite trend compared to short-term infected and healthy fruits [36,37]. In necrotic tissue, fructose remained stable over all the study period [37]. A significant decrease in sorbitol was only recorded for infected fruits [36].

In conclusion, in some cases, infected fruits had fewer total sugars. Ripening changed the sugar composition, but PPV infection altered this mechanism. The duration of infection significantly influenced both the ripening and sugar compositions. Overall, the results observed for the studies on sugars showed a greater number of similarities between short-term infected and healthy fruits than between long-term and necrotic tissues, which showed an altered composition for individual sugars.

#### 4.2.3. Organic Acids: Content and Composition

In general, the relative content of organic acids decreased during ripening. Nonetheless, in both short-term and long-term infected trees, the relative content of organic acids decreased early [36,37]. At day 0, fruits from long-term infected trees had significantly lower organic acid contents. At day 22, fruits from healthy trees had significantly higher organic acids contents than infected trees, but between day 22 and day 30, organic acids decreased significantly until the fruits were fully ripened [36,37]. In apricots, the only available data showed a significant increase in organic acids in symptomatic fruits [39].

The predominant organic acid in plum and apricot fruits was malic acid, the key contributor to the total sugar/organic acid ratio (TS/OA) [37,39]. The malic acid content was altered by PPV infection, and necrotic tissue had the lowest relative content, especially on day 22 [37]. In another study conducted by Usenik et al. [36], long-term infected fruit had the lowest content of malic acid compared to short-term infected and healthy fruits. The citric acid content was significantly lower in long-term infected fruits compared to healthy fruits [36], but the highest content was found in necrotic tissue for all sampling dates [37]. Moreover, the shikimic acid content of long-term infected fruits was significantly lower compared to short-term infected and healthy fruits.

In general, PPV infection causes an earlier ripening of fruits. This was confirmed by the early decrease in organic acids in infected fruits. Furthermore, the duration of the infection significantly modified the organic acid content. In the case of organic acids, long-term and short-term infected trees are more similar and could be differentiated from healthy trees [36].

#### 4.2.4. Quality Fruit Indexes

The ripening index (RI or SSC/TA ratio) has an important role in consumer acceptance of fruits. A high RI ratio reveals a good fruit quality and taste. Uninfected fruits had a higher RI compared to infected PPV fruits due to lower acidity [39]. Fruits with high total sugar contents (TS) and low total organic acids (TA) are considered as sweet fruits. In contrast, a low value for the TS/TA ratio indicates a sour taste. The TS/TA ratio increased earlier in both infected trees [36]. Nevertheless, the ratio increased the most in healthy and short-term infected fruits for plums and apricots [37,39]. The lowest ratio was found in necrotic tissue [37].

PPV infection altered the maturation process. Long-term infected fruits had a lower ripening index [39] and a sour taste and were more acidic than fully ripened healthy fruits on day 30 [37,38,40]. Premature fruit dropping thus yields fruits with an inferior taste for both plums and apricots.

### 4.3. Secondary Compounds: The Case of Phenolics

Two main studies explored the modification of phenolic compounds induced by PPV infection in plums [36,38]. In both studies, the effect of PPV-Rec on healthy versus infected trees was studied during the last 3 weeks of ripening. Fruits were hand-picked on three sampling dates at days 0, 9, and 22. Usenik et al. [36] also sampled healthy trees at day 30 and conducted an experiment with infected trees that displayed different durations of infection: short-term infected trees (at least 1 year) versus long-term infected trees (at least 5 years). In the other study, Usenik et al. [38] analyzed infected trees with a unknown duration of infection by using visually undeformed and necrotic fruit tissues. Nevertheless, infected trees displayed similar symptoms to long-term infected trees, with strong symptoms on the leaves and fruits and a similar level of premature fruit dropping.

In these studies, three main groups of phenolics were analyzed in the edible part of plum fruits: anthocyanins, flavonols, and hydroxycinnamic acids. Hydroxycinnamic acids represented the predominant group of phenolics, followed by anthocyanins and flavonols [36,38]. The contents and compositions of the phenolic compounds in plum fruits were significantly modified by the PPV infection. The duration of PPV infection or the type of tissue had an important influence. The phenolic content and composition were highly altered in long-term infected trees and necrotic tissues [36,38]. In contrast, in apricots, the total phenolic and flavonoid contents were lower in infected fruits. These opposite results could be explained by the mixed PPV infection (PPV-Rec + PPV-D) or the ripening influences on dynamic modifications [39]. In general, the PPV promoted an increase in phenolic compounds, particularly for anthocyanins and flavonols, leading to a reduced synthesis of hydroxycinnamic acids. The alteration in the composition of phenolics is only one of the symptoms of PPV infection and is the consequence of a defense response against the pathogen [38].

#### 4.3.1. Hydroxycinnamic Acids: Content and Composition

During ripening, while the relative content of hydroxycinnamic acid (HCA) decreased, in infected fruits, the content was significantly lower in both undeformed and necrotic tissues [38]. In another study, Usenik et al. [36] showed that the HCA content in long-term infected trees exhibited a similar trend. In short-term infected fruits, the HCA content was higher than healthy fruits at day 0 but decreased significantly during ripening to levels below those found in healthy fruits but higher than those found in long-term infected fruits.

Usenik et al. [36,38] analyzed the HCA composition in fruits. Four derivates were identified in both studies: the predominant neochlorogenic acid (NA), chlorogenic acid (CA), *p*-coumaroylquinic acid (PCA), and cryptochlorogenic acid (CRY). The average content of NA and PCA was influenced only by PPV infection, provoking a lower content of NA and a higher content of PCA in infected fruits, with the highest levels found in necrotic tissue [38]. Usenik et al. [36] reported a similar trend for both compounds in long-term infected fruits. Concerning short-term infected fruits, the NA content decreased significantly during ripening until day 22 and was less than that found for healthy fruits. The content levels of CA depended on the tissue type and ripening stage. Only necrotic tissue showed a significant modified CA content, where modifications occurred between day 0 and day 9 [38]. In long-term infected fruits, the CA content was lower than short-term infected and healthy fruits [36]. For the CRY content, no significant differences were detected, except for necrotic tissues [36,38].

NA, PCA, and CA compounds could be used to discriminate between healthy and long-term infected trees. For short-term infected trees, only the NA compound could discriminate between healthy and contaminated trees. However, the alteration of this compound is influenced by both infection and maturation.

#### 4.3.2. Anthocyanins: Content and Composition

Anthocyanins (ANTH) are involved in the internal regulation of plant cell physiology and signaling, and they act as general antioxidants in various stress situations and are also responsible for the color changes associated with ripening [36,38]. Fruits accumulate ANTH during ripening, but the relative content was significantly higher in infected fruits in both studies, independent of the time of infection and tissue type. However, the highest content of ANTH was reported in long-term infected fruits [36] and in necrotic tissue [38]. These results were consistent with the visual appearance and the measurement of skin color in fruits. The higher ANTH content in the infected fruits showed the stress-protective role of ANTH in the plant. The following anthocyanins were identified: cyanidin-3-O-glucoside (CG), cyanidin-3-O-rutinoside (CR), peonidin-3-O-glucoside (PG), and peonidin-3-O-rutinoside. CR was identified as the main ANTH in *Prunus domestica* plums [36,38]. The relative concentration of all ANTH increased with time for all tissue types.

The content of both cyanidin compounds, CG and CR, was higher in infected fruit from day 9 to day 22 in both long- and short-term infected trees [36] and tissue type (undeformed and necrotic tissues) [38]. However, the content was higher in necrotic tissue [38] and in long-term infected fruits and increased earlier, starting from day 0 [36]. In healthy fruits, the content of both compounds increased only after day 22 but remained lower than infected trees at day 22 [36]. Usenik et al. [36,38] also reported that the content of PG, which increased with time, was significantly higher in infected fruits, with a higher content in necrotic tissue and long-term infected trees. The content of peonidin-3-O-rutinoside was also altered by PPV infection. Usenik et al. [36] demonstrated that this compound was lowest in long-term infected fruits, whereas Usenik et al. [38] reported that undeformed tissues from infected fruits shared similarities with healthy fruits.

There are significant differences in the composition of anthocyanins between PPV-infected and healthy fruits. These differences are more important in long-term infected fruits and necrotic tissue than short-term infected and healthy fruits [36,38]. Here, the short-term infected fruits shared more similarities with long-term infected trees [36]. CG, CR, and PG compounds are significantly altered by PPV infection in both long- and short-term infected trees. Nevertheless, the ripening process could impact the levels of these compounds, as observed at day 0—in particular, for short-term infected trees. These are potential biomarkers of the sharka disease; however, further investigations are needed to prove this hypothesis.

#### 4.3.3. Flavonols: Content and Composition

The relative flavonols content was significantly higher in infected fruits and increased with ripening, particularly in long-term infected fruits and in necrotic tissues [36,38]. According to these authors, flavonols may have a protective role against stress in PPV infection, and in particular, they analyzed, rutin (RU), quercetin-3-O-galactoside (QGA), quercetin-3-O-glucoside (QGL), kaempferol-3-O-rutinoside (KR), and isorhamnetin-3-O-rutinoside (IR). The greatest increase due to infection was monitored in the QGL compound; its level significantly increased with ripening and was the highest in infected fruits, especially in those with long-term infection [36,38]. The long-term infected fruits from Usenik et al. [36] and infected fruits from Usenik et al. [38] had significantly more RU and QGA, with the highest levels found in necrotic tissue. PPV infection had no significance on the KR and IR contents in infected fruits [36] but was only significantly higher in necrotic tissue [38].

Here, RU, QGA, and QGL could be applied to discriminate between long-term infected and healthy trees. Discrimination between short-term infected and healthy trees may be possible, but ripening may have a significant effect on these metabolites.

## 5. Metabolomics: An Insightful Tool to Study *Prunus*—PPV Interactions?

Metabolomics is a tool for the comprehensive and systematic identification and quantification of metabolites with small molecular weights (<1500 Daltons) in biological samples at a given point in time. The metabolome represents a set of all metabolites present in cells, tissues, organs, or organisms. This approach can reveal the dynamic physiological conditions that correspond to the impact of a disease on the host. The use of metabolomics in the host/pathogen interaction leads to the discovery of new biomarkers for the prognosis and/or diagnostic of diseases, such as obesity or diabetes [41].

Currently, the use of metabolomics is expanding into new areas, and this technology is emerging as a promising tool for the study of plant–pathogen interactions. Generally, a metabolomics analysis is performed by nuclear magnetic resonance (NMR) spectroscopy and mass spectrometry (MS) coupled with liquid chromatography (LC) or gas chromatography (GC). The nontargeted approach, which is a qualitative analysis, provides a global profile for many unknown metabolites in a sample, whereas the targeted approach, which is a quantitative analysis, is more specific and is aimed at a defined class of known compounds [8]. Castro-Moretti et al. [8] showed that pathogen infections altered the primary and secondary metabolisms. Compounds involved in the primary metabolism are crucial to growth, development, and reproduction in plants, while the secondary metabolism includes the compounds necessary for plants to respond successfully to abiotic and biotic stresses. A comparison of infected and healthy plants using a nontargeted approach would provide insight into the pathways and/or metabolites affected by the infection, even before the onset of symptoms. Therefore, the elucidation of these metabolic modifications may be useful for the identification of infected trees. These candidate biomarkers could be used to reveal disease, even at an early stage. For instance, Asteggiano et al. [42] developed a global metabolomics approach for the diagnostic of “Olive Quick Decline Syndrome” in olive tree leaves. In addition, Garcia et al. [43] developed a combined metabolomics approach to detect blight-infected tomato plants. This detection could even indicate the stages of infection in asymptomatic leaves. Tomatidine is proposed as the major metabolic biomarker of infection. In addition, the authors determined that, for asymptomatic leaves, saponins may allow for the early detection of disease, while isocoumarin may allow for the detection of disease at late stages of infection.

In the case of *Prunus*–PPV interactions, the studies presented in this review reveal the impact induced by sharka disease in *Prunus* species. The set of changes induced by PPV infection observed in leaves, as well as in fruits, could provide many clues for the development of a new methodological approach. Differences were observed at several levels within the chloroplasts, the antioxidant system, the phytohormone profile, the transcriptome, the proteome, and even the translatome in *Prunus* leaves. In addition, sugars, organic acids, and phenolic compounds were also altered by PPV infection in *Prunus* fruit. In general, most of the changes produced by PPV infection are related to photosynthesis, defense responses, and primary (sugars and organic acids) and secondary (phenolic compounds) metabolisms.

Interestingly, the results obtained by Rubio et al. [20] and Collum et al. [25] highlighted the existence of early responses to the onset of symptoms in *Prunus* leaves. Rubio et al. [20] revealed early responses to PPV infection in asymptomatic leaves, associated with the induction of genes related to defense responses such as *chitinases* and SAM *synthetase*. In addition, *SAM synthetase* was only overexpressed in asymptomatic leaves. The responses identified in asymptomatic leaves can be considered as early responses to the onset of PPV symptoms. In addition to these findings, Collum et al. [25] highlighted the temporal and spatial coordination of defense responses in phloem tissues. During the early stages of infection (4- and 6-week-old leaves), the phloem tissues had a disproportionate response compared to non-phloem tissues. For instance, genes related to PR protein and receptor kinases were overexpressed at 4 weeks post-cold-induced dormancy only in phloem tissues. Moreover, the first symptoms appeared by 6 weeks post-cold-induced dormancy. Therefore, all of the induced changes up to this time point can also be considered as early responses to the onset of PPV symptoms. A further study could identify the responses that may allow for the early detection of PPV-infected trees. Together, these results suggest that it may now be relevant to use metabolomics to characterize these types of responses in order to identify an early response in planta.

In addition to early responses, some genes appear to be present in both symptomatic and asymptomatic leaves. The findings provided by Rubio et al. [20] suggested a new perspective for investigation. In fact, a comparison between symptomatic and asymptomatic leaves from the same plant revealed the existence of different patterns at the transcriptomic level. However, some genes were overexpressed in both symptomatic and asymptomatic peach leaves, including *allene oxide synthase*, the transcription factor *bHLH*, and *chitinases*. These results suggest that a systemic response to PPV infection may exist. No other studies that compared symptomatic and asymptomatic samples from the same plant were found. Further investigation and identification of systemic responses should be pursued. A metabolomics analysis could reveal the existence of a systemic response to PPV infection by comparing asymptomatic, symptomatic, and healthy samples. This theoretical systemic response could allow for the development of a method for the rapid detection of PPV-infected trees, even in asymptomatic samples. The key factor to consider in this review is that the focus is not on PPV detection but, instead, on the metabolic changes induced by PPV infection in planta. The results presented above provide important clues for future research. First, the early responses to PPV infection highlighted at the transcriptome and translatome levels could be explored for the early detection of infected trees. Detection before the onset of PPV symptoms would be a key tool for controlling the spread of sharka disease. Second, the existence of a systemic response to PPV infection could be exploited to develop a new methodology. This would allow for the identification of PPV-infected *Prunus*, whether in symptomatic or asymptomatic leaves. The development of this approach could promote the rapid and large-scale detection of contaminated trees and, therefore, would help to control the spread of the virus. It should be noted that the analyses described in the previous sections concerning leaves and fruit were performed only on one tissue type in each case. It would therefore be interesting to perform a metabolomics analysis on leaves and fruits to acquire a comprehensive knowledge of the disease. Furthermore, as described earlier, the response to PPV infection could be modified by *Prunus* species and/or cultivar. Therefore, a species and/or cultivar-specific analysis might be necessary for biomarker detection. One would expect that there would be different types of responses between species but, also, common biomarkers between species. The multi-omics analyses described in this review contribute to the understanding of *Prunus*–PPV interactions and reveal the complex and dynamic responses of *Prunus* to PPV infection. However, for a better understanding of these interactions, it is important to study how the metabolome is altered by infection. As previously mentioned, metabolomics has the power to highlight the impact produced by the pathogen in plants. In addition, a nontargeted approach can identify biomarkers related to the infection. This approach has been successfully applied in the detection of plant diseases. Therefore, an untargeted metabolomics analysis is an interesting approach to investigate: (i) the global *Prunus*–PPV interactions, (ii) the early responses before sharka symptoms, and (iii) the systemic *Prunus* responses to PPV infection.

Nevertheless, several parameters could limit the development of a metabolomics-based method for detecting infected trees. A global analysis of the metabolome, through nontargeted approaches, frequently requires a set of different analytical tools (LC-MS, GC-MS, and RMN), which can be expensive. Furthermore, candidate biomarkers need to be identified, quantified, and validated before being selected for diagnostic or prognosis purposes. For metabolite identification and quantification, targeted approaches are used [44]. The validation step is also complex and can be challenging, as it requires the evaluation of several parameters, such as the limit of detection (LOD), quantitation (LOQ), linearity (R^2^), linear range, and repeatability. Finally, the biomarkers must be confirmed in a different set of samples [45]. Once the biomarkers are validated, they can be used for diagnostic or prognosis purposes by targeted approaches that, in general, are cheaper and faster than untargeted methods. In addition, a full instrumental platform is not required for this purpose, which would simplify the application of the routine diagnostic [42]. However, several conditions must be provided to ensure routine tests. To simplify the assay, the analysis should be performed with a small number of biomarkers—ideally, 1–10 metabolites maximum. Keep in mind that a key step for large-scale analyses requires a reduced number of biomarkers, thus reducing the time and cost of workflow analyses. Moreover, new LC coupled to tandem mass spectrometry (LC-MS/MS) systems are currently available to maximize the productivity in diagnostic applications [44].

## 6. Conclusions and Perspectives

This review summarized the impact of PPV infection in *Prunus* of the leaves and fruit tissues. Most of the changes produced by PPV infection are related to photosynthesis, defense responses, and the primary and secondary metabolisms. Moreover, some authors highlighted that these changes were complex and dynamic. Interestingly, many results from the literature suggested the presence of systemic and early responses. These features could be used to develop a new methodology for the detection of infected trees by a metabolomics approach. Nevertheless, many parameters could influence the results, such as the *Prunus* species or varieties, PPV strain, the presence or absence of symptoms, the type of tissue, the duration of infection, and the ripening process. Metabolomics is described as an emerging tool for the study of plant–pathogen interactions and can provide, through an untargeted analysis, a global profile for infected and healthy plants. Comparisons between infected and healthy plants would allow for the identification of biomarkers related to PPV infection. Some studies mentioned in this paper highlighted the application of metabolomics to develop detection tools. A metabolomics approach seems to be a suitable option for investigating potential systemic, as well as early, plant responses in reaction to PPV infection. The detection and identification of biomarkers implicated in these kinds of responses could be used for the development of a targeted method for the detection of infected trees. The major advantage would be the reliable and early detection of infected trees, which would allow better monitoring of a PPV outbreak.

For example, in plum fruits, some phenolic compounds, including neochlorogenic acid, cyanidin-3-O-glucoside and cyanidin-3-O-rutinoside, peonidin-3-O-glucoside, rutin, and quercetin-3-O-galactoside and quercetin-3-O-glucoside, analyzed by a targeted approach appear to discriminate between healthy and PPV-infected trees. These compounds need to be further investigated in a large experiment to be confirmed as candidate biomarkers for sharka disease and to assess whether these could lead to early detection. However, these compounds could be part of a generic response to several viral diseases and not necessarily specific to sharka disease.

## References

[1] García J.A., Glasa M., Cambra M., Candresse T. (2014). Plum pox virus and sharka: A model potyvirus and a major disease. Mol. Plant Pathol..

[2] Rodamilans B., Valli A., García J.A. (2020). Molecular Plant-Plum Pox Virus Interactions. Mol. Plant Microbe Interact..

[3] Rimbaud L., Dallot S., Gottwald T., Decroocq V., Jacquot E., Soubeyrand S., Thébaud G. (2015). Sharka Epidemiology and Worldwide Management Strategies: Learning Lessons to Optimize Disease Control in Perennial Plants. Annu. Rev. Phytopathol..

[4] Ilardi V., Tavazza M. (2015). Biotechnological strategies and tools for Plum pox virus resistance: Trans-, intra-, cis-genesis, and beyond. Front. Plant Sci..

[5] Rimbaud L., Dallot S., Bruchou C., Thoyer S., Jacquot E., Soubeyrand S., Thébaud G. (2019). Improving Management Strategies of Plant Diseases Using Sequential Sensitivity Analyses. Phytopathology.

[6] FAO (2012). ISPM 27 Diagnostic Protocols for Regulated Pests, DP 2: Plum pox virus.

[7] Rimbaud L., Dallot S., Delaunay A., Borron S., Soubeyrand S., Thébaud G., Jacquot E. (2015). Assessing the mismatch between incubation and latent periods for vector-borne diseases: The case of sharka. Phytopathology.

[8] Castro-Moretti F.R., Gentzel I.N., Mackey D., Alonso A.P. (2020). Metabolomics as an Emerging Tool for the Study of Plant–Pathogen Interactions. Metabolites.

[9] Hernandez J.A., Rubio M., Olmos E., Ros-Barcelo A., Martinez-Gomez P. (2004). Oxidative stress induced by long-term plum pox virus infection in peach (*Prunus persica*). Physiol. Plant..

[10] Clemente-Moreno M.J., Díaz-Vivancos P., Rubio M., Fernández-García N., Hernández J.A. (2013). Chloroplast protection in plum pox virus-infected peach plants by L-2-oxo-4-thiazolidine-carboxylic acid treatments: Effect in the proteome. Plant. Cell Environ..

[11] Clemente-Moreno M.J., Hernández J.A., Diaz-Vivancos P. (2015). Sharka: How do plants respond to Plum pox virus infection?. J. Exp. Bot..

[12] Hernandez J.A., Diaz-Vivancos P., Rubio M., Olmos E., Ros-Barcelo A., Martinez-Gomez P. (2006). Long-term plum pox virus infection produces an oxidative stress in a susceptible apricot, Prunus armeniaca, cultivar but not in a resistant cultivar. Physiol. Plant..

[13] Murchie E.H., Lawson T. (2013). Chlorophyll fluorescence analysis: A guide to good practice and understanding some new applications. J. Exp. Bot..

[14] Hasanuzzaman M., Bhuyan M.H.M.B., Zulfiqar F., Raza A., Mohsin S.M., Al Mahmud J., Fujita M., Fotopoulos V. (2020). Reactive Oxygen Species and Antioxidant Defense in Plants under Abiotic Stress: Revisiting the Crucial Role of a Universal Defense Regulator. Antioxidants.

[15] Clemente-Moreno M.J., Piqueras A., Hernández J.A. (2011). Implication of peroxidase activity in development of healthy and PPV-infected micropropagated GF305 peach plants. Plant Growth Regul..

[16] Diaz-Vivancos P., Rubio M., Mesonero V., Periago P., Ros Barcelo A., Martinez-Gomez P., Hernandez J. (2006). The apoplastic antioxidant system in Prunus: Response to long-term plum pox virus infection. J. Exp. Bot..

[17] Hernández J., Díaz-Vivancos P., Rubio M., Olmos E., Clemente M., Ros-Barceló A., Martínez-Gómez P. (2007). Plum pox virus (PPV) infection produces an imbalance on the antioxidative systems in Prunus species. Acta Phytopathol. Entomol. Hung..

[18] Hernández J.A., Talavera J.M., Martínez-Gómez P., Dicenta F., Sevilla F. (2001). Response of antioxidative enzymes to plum pox virus in two apricot cultivars. Physiol. Plant..

[19] Dehkordi A., Rubio M., Babaeian N., Albacete A., Martínez-Gómez P. (2018). Phytohormone Signaling of the Resistance to Plum pox virus (PPV, Sharka Disease) Induced by Almond (*Prunus dulcis* (Miller) Webb) Grafting to Peach (*P. persica* L. Batsch). Viruses.

[20] Rubio M., Rodríguez-Moreno L., Ballester A.R., de Moura M.C., Bonghi C., Candresse T., Martínez-Gómez P. (2015). Analysis of gene expression changes in peach leaves in response to Plum pox virus infection using RNA-Seq. Mol. Plant Pathol..

[21] Clemente-Moreno M.J., Díaz-Vivancos P., Piqueras A., Hernández J.A. (2012). Plant growth stimulation in Prunus species plantlets by BTH or OTC treatments under in vitro conditions. J. Plant Physiol..

[22] Wang A., Chapman P., Chen L., Stobbs L.W., Brown D.C.W., Brandle J.E. (2005). A comparative survey, by expressed sequence tag analysis, of genes expressed in peach leaves infected with Plum pox virus (PPV) and free from PPV. Can. J. Plant Pathol..

[23] Rubio M., Ballester A.R., Olivares P.M., De Moura M.C., Dicenta F., Martínez-Gómez P. (2015). Gene expression analysis of plum pox virus (Sharka) susceptibility/resistance in apricot (*prunus armeniaca* L.). PLoS ONE.

[24] Zuriaga E., Romero C., Blanca J.M., Badenes M.L. (2018). Resistance to Plum Pox Virus (PPV) in apricot (*Prunus armeniaca* L.) is associated with down-regulation of two MATHd genes. BMC Plant Biol..

[25] Collum T.D., Stone A.L., Sherman D.J., Rogers E.E., Dardick C., Culver J.N. (2020). Translatome Profiling of Plum Pox Virus–Infected Leaves in European Plum Reveals Temporal and Spatial Coordination of Defense Responses in Phloem Tissues. Mol. Plant Microbe Interact..

[26] King H.A., Gerber A.P. (2014). Translatome profiling: Methods for genome-scale analysis of mRNA translation. Brief. Funct. Genom..

[27] Collum T.D., Lutton E., Raines C.D., Dardick C., Culver J.N. (2019). Identification of phloem-associated translatome alterations during leaf development in *Prunus domestica* L.. Hortic. Res..

[28] Bernal-Vicente A., Cantabella D., Hernández J.A., Diaz-Vivancos P. (2018). The effect of mandelonitrile, a recently described salicylic acid precursor, on peach plant response against abiotic and biotic stresses. Plant Biol..

[29] Alazem M., Lin N. (2015). Roles of plant hormones in the regulation of host–virus interactions. Mol. Plant Pathol..

[30] Rubio J., Sánchez E., Tricon D., Montes C., Eyquard J.-P., Chague A., Aguirre C., Prieto H., Decroocq V. (2019). Silencing of one copy of the translation initiation factor eIFiso4G in Japanese plum (*Prunus salicina*) impacts susceptibility to Plum pox virus (PPV) and small RNA production. BMC Plant Biol..

[31] Decroocq V., Sicard O., Alamillo J.M., Lansac M., Eyquard J.P., García J.A., Candresse T., Le Gall O., Revers F. (2006). Multiple Resistance Traits Control Plum pox virus Infection in Arabidopsis thaliana. Mol. Plant Microbe Interact..

[32] Wang X., Kohalmi S.E., Svircev A., Wang A., Sanfaçon H., Tian L. (2013). Silencing of the Host Factor eIF(iso)4E Gene Confers Plum Pox Virus Resistance in Plum. PLoS ONE.

[33] Rodamilans B., San León D., Mühlberger L., Candresse T., Neumüller M., Oliveros J.C., García J.A. (2014). Transcriptomic analysis of Prunus domestica undergoing hypersensitive response to Plum Pox Virus infection. PLoS ONE.

[34] Markiewicz M., Michalczuk L., Neumüller M. (2019). Hypersensitive reaction of plum (*Prunus domestica*) in response to Plum pox virus infection: Changes in gene expression and identification of potential molecular markers. Sci. Hortic..

[35] Milosevic T.M., Glisic I.P., Milosevic N.T., Glisic I.S. (2010). Plum pox virus as a stress factor in the vegetative growth, fruit growth and yield of plum (*Prunus domestica*) cv. ‘Cacanska Rodna’. Eur. J. Plant Pathol..

[36] Usenik V., Kastelec D., Stampar F., Virscek Marn M. (2015). Effect of Plum pox virus on Chemical Composition and Fruit Quality of Plum. J. Agric. Food Chem..

[37] Usenik V., Marn M.V. (2017). Sugars and organic acids in plum fruit affected by Plum pox virus. J. Sci. Food Agric..

[38] Usenik V., Franci S., Damijana K. (2017). How does sharka affect the phenolics of plum fruit (*Prunus domestica* L.)?. Hortic. Sci..

[39] Milošević T., Milošević N., Mladenović J., Jevremović D. (2019). Impact of Sharka disease on tree growth, productivity and fruit quality of apricot (*Prunus armeniaca* L.). Sci. Hortic..

[40] Samara R., Hunter D.M., Stobbs L.W., Greig N., Lowery D.T., Delury N.C. (2017). Impact of Plum pox virus (PPV-D) infection on peach tree growth, productivity and bud cold hardiness. Can. J. Plant Pathol..

[41] Cheng D., Zhao X., Yang S., Cui H., Wang G. (2021). Metabolomic Signature Between Metabolically Healthy Overweight/Obese and Metabolically Unhealthy Overweight/Obese: A Systematic Review. Diabetes Metab. Syndr. Obes. Targets Ther..

[42] Asteggiano A., Franceschi P., Zorzi M., Aigotti R., Dal Bello F., Baldassarre F., Lops F., Carlucci A., Medana C., Ciccarella G. (2021). HPLC-HRMS Global Metabolomics Approach for the Diagnosis of “Olive Quick Decline Syndrome” Markers in Olive Trees Leaves. Metabolites.

[43] Galeano Garcia P., Neves dos Santos F., Zanotta S., Eberlin M., Carazzone C. (2018). Metabolomics of Solanum lycopersicum Infected with Phytophthora infestans Leads to Early Detection of Late Blight in Asymptomatic Plants. Molecules.

[44] Pinu F.R., Goldansaz S.A., Jaine J. (2019). Translational Metabolomics: Current Challenges and Future Opportunities. Metabolites.

[45] Ahonen L., Jäntti S., Suvitaival T., Theilade S., Risz C., Kostiainen R., Rossing P., Orešič M., Hyötyläinen T. (2019). Targeted Clinical Metabolite Profiling Platform for the Stratification of Diabetic Patients. Metabolites.

